# Antagonistic effect of *Lactobacillus *strains against gas-producing coliforms isolated from colicky infants

**DOI:** 10.1186/1471-2180-11-157

**Published:** 2011-06-30

**Authors:** Francesco Savino, Lisa Cordisco, Valentina Tarasco, Emanuela Locatelli, Diana Di Gioia, Roberto Oggero, Diego Matteuzzi

**Affiliations:** 1 Department of Pediatrics, Regina Margherita Children Hospital, P.zza Polonia 94, Torino, 10126, Italy; 2 Department of Pharmaceutical Sciences, University of Bologna, via Belmeloro 6, Bologna, 40126, Italy; 3 Department of Agroenvironmental Sciences and Technologies - Microbiology Area, University of Bologna, Via Fanin 42, 40127, Bologna, Italy

## Abstract

**Background:**

Infantile colic is a common disturb within the first 3 months of life, nevertheless the pathogenesis is incompletely understood and treatment remains an open issue. Intestinal gas production is thought to be one of the causes of abdominal discomfort in infants suffering from colic. However, data about the role of the amount of gas produced by infants' colonic microbiota and the correlation with the onset of colic symptoms are scanty. The benefit of supplementation with lactobacilli been recently reported but the mechanisms by which they exert their effects have not yet been fully defined. This study was performed to evaluate the interaction between *Lactobacillus *spp. strains and gas-forming coliforms isolated from stools of colicky infants.

**Results:**

Strains of coliforms were isolated from stools of 45 colicky and 42 control breastfed infants in McConkey Agar and identified using PCR with species-specific primers, and the BBL™ Enterotube™ II system for *Enterobacteriaceae*. Gas-forming capability of coliforms was assessed in liquid cultures containing lactose as sole carbon source. The average count of total coliforms in colicky infants was significantly higher than controls: 5.98 (2.00-8.76) log_10 _*vs *3.90 (2.50-7.10) CFU/g of faeces (p = 0.015). The following strains were identified: *Escherichia coli*, *Klebsiella pneumoniae*, *Klebsiella oxytoca*, *Enterobacter aerogenes*, *Enterobacter cloacae *and *Enterococcus faecalis*. Then, 27 *Lactobacillus *strains were tested for their antagonistic effect against coliforms both by halo-forming method and in liquid co-cultures. *Lactobacillus delbrueckii *subsp.*delbrueckii *DSM 20074 and *L. plantarum *MB 456 were able to inhibit all coliforms strains (halo-forming method), also in liquid co-cultures, thus demonstrating an antagonistic activity.

**Conclusions:**

This study shows that two out of 27 strains of *Lactobacillus *examined possess an antimicrobial effect against six species of gas-forming coliforms isolated from colicky infants. Our findings may stimulate new researches to identify which *Lactobacillus *strains can improve colicky symptoms by acting on coliforms gut colonization.

## Background

The intestinal microbiota exerts many physiological functions such as metabolic and trophic activities and plays an important role in the "barrier effect" against exogenous microbes [1]. It is also involved in the development and activation of the intestinal immune system: a recent study suggests that a more diverse gut microbiota early in life might prevent allergy development [2]. Gut microbiota is acquired during early life and intestinal colonization starts immediately after birth. The ability of species to establish themselves durably in the colonic ecosystem depends on complex interactions between host and bacteria as well as between the bacteria themselves [3]. A wide range of factors may influence the establishment of the intestinal microbiota, including type of delivery, feeding pattern, antibiotic therapy, contact with parents, siblings and hospital staff [4]. The nature of the gut flora, colonic bacterial metabolic pathways, the partial pressure of hydrogen in the colon, the buffering capacity of the colon, and incomplete monosaccharide absorption may play a part in infantile colic. Miller reported an increased breath hydrogen excretion in subjects suffering from infantile colic [5]. In 1994, Lehtonen *et al. *observed that an inadequate lactobacilli level occurring in the first months of life may affect the intestinal fatty acids profile and could favour the development of infantile colic [6]. Treem suggested that colicky infants produce large amounts of gas probably by colonic bacterial fermentation of malabsorbed dietary carbohydrate and that they are relieved of symptoms by the passage of gas [7]. It has also been demonstrated that less methane is produced by stool of colicky infants and this could be due to an inability of the gut microbiota to convert hydrogen to methane with a gastrointestinal hydrogen accumulation [8]. Moreover few old data support the notion that colicky infants produce more breath hydrogen in the fasting state and in response to feedings, which is thought to be evidence of lactose intolerance [9-11].

Differences in gut microbiota have been found among colicky and non-colicky infants: colicky infants are less frequently colonized by *Lactobacillus *spp. and more frequently by anaerobic gram-negative bacteria [12]. Further, different colonization patterns of lactobacilli have been found among colicky and healthy infants: *L. brevis *and *L. lactis *are present only in colicky infants while *L. acidophilus *was detected only in healthy ones [13].

The recent finding that *L. reuteri *improve colic symptoms in breastfed infants suggested that a peculiar composition of the intestinal microbiota could favour the development of such disturbance [14,15]; however the mechanisms through which lactic acid bacteria act on colic symptoms remain speculative. Moreover, our recent study evaluated the colonization pattern of some important gas-forming coliforms in colicky infants and healthy controls through molecular methods: coliform bacteria, in particular *Escherichia coli*, resulted more abundant in infants with infantile colic, reinforcing the concept that gut microbiota could be implicated in the aetiopathogenesis of the disturbance [16,17]. Nevertheless, up today, little is known about the role of the amount of gas produced by infants' colonic microbiota and the correlation with the onset of colic symptoms, even thought intestinal gas is though to be one of the causes of abdominal discomfort.

This study was performed to elucidate the interaction between lactobacilli and gas-forming coliforms in the gut. To this aim, 27 *Lactobacillus *strains were examined for their potential *in-vitro *anti-microbial activity against gas-forming coliforms isolated from stools of colicky infants.

## Methods

### Study group and sample collection

Forty-five breastfed infants suffering from colic symptoms and 42 control breastfed infants (i.e. non colicky) were recruited at the Department of Pediatrics - Regina Margherita Children Hospital, Turin, Italy. They were all aged between 4 and 12 weeks, adequate for gestational age, with a birth weight in the range 2500 and 4000 g, without clinical evidence of chronic illness or gastrointestinal disorders or previous administration of antibiotics and probiotics in the week preceding recruitment. The characteristics of colicky and control subjects are shown in Table 1. Only exclusively breastfed infants were enrolled in order to reduce variability in the intestinal microflora and in the colonic gas associated with dietary variations [18,19]. The colicky cry was defined as a distinctive pain cry difficult to console, lasted for 3 hours or more per day on 3 days or more per week, diagnosed according Wessel criteria [20], with debut 6 ± 1 days before the enrolment. At the enrolment each subject underwent a medical examination and parents were interviewed in order to obtain background data concerning type of delivery, birth weight and gestational age, family history of gastrointestinal disease and atopy. Parents gave written consent to the inclusion of their infants in the study. About 5-10 g faeces were collected from both colicky and non-colicky infants, stored at - 80°C immediately after collection and subsequently processed. The study was approved by the local ethic committee (Comitato Interaziendale AA.SS.OO. O.I.R.M./S. Anna-Ordine Mauriziano di Torino).

**Table 1:** Clinical characteristics of the study population and count of total coliforms bacteria

	Colicky infants (n = 45)	Controls (n = 42)	p-value
Gender (M/F)	25/20	24/18	1.000**
Age at recruitment (days)	42 (15-95)	39 (17-98)	0.788*
Type of delivery (spontaneous/caesarean)	27/18	23/19	0.668**
Birth weight (grams)	3300 (2550-3970)	3350 (2520-4010)	0.951*
Crying time (minutes per day)	225 (185-310)	105 (60-135)	0.000*
Average count of total coliform bacteria (log10 CFU/g of faeces)	5.98 (2.00-8.76)	3.90 (2.50-7.10)	0.015*

Data are expressed as median (range) or numbers.

*Mann-Whitney Test.

**Fisher's Exact Test

### Isolation and identification of coliforms

Faecal samples, collected from all infants, were homogenized (10%, w/v) with sterile saline (0.9% NaCl). The homogenates were filtered through a 100 μm metal sieve and serially diluted in saline. One hundred μl of each dilution were plated on selective MacConkey Agar (BD Italia, Milan, Italy), which is widely used to isolate enteric bacteria and as a presumptive test for coliform organisms [21] and plates were incubated overnight at 37°C in 5% CO2 atmosphere. All colonies were counted and counts expressed as log10 colony-forming units (CFU) per g of faeces. Each isolated strain was subcultured at 37°C for 18 h in Luria Bertani medium (LB) [22] under microaerophilic conditions. Identification of the isolated strains was performed by using the polymerase chain reaction (PCR) technique followed by sequencing of the amplified sequences and the BBL™ Enterotube™ II system, which allows the identification of *Enterobacteriaceae *on the basis of selective carbohydrate fermentation, gas production and the response to selective biochemical reactions (Becton Dickinson GmbH, Heidelberg, Germany). PCR was performed as follows: each isolated strain was streaked on a LB plate, which was incubated overnight at 37°C. A single colony of each strain was picked and suspended in 20 μl of sterile distilled water; the cell suspension was heated at 95°C for 10 min and then cooled to 4°C. The rDNA fragment comprising the internal transcribed spacer and the flanking 16S and 23S rDNA regions was amplified by using the primers indicated in a previous paper [17] and a Biometra (M-Medical SrL, Milan, Italy) thermocycler; the amplified fragments were sequenced and aligned with the most similar ones of GenBank using the Basic Local Alignment Search Tool (BLAST) program.

### Evaluation of the gas-forming capability of the isolated strains

The gas-forming capability of the strains isolated from stool samples was assessed in Lauryl sulphate tryptose broth containing lactose (10 g/L) as the sole carbon source. After inoculum and incubation for 24-48 h at 37°C, bacterial cultures were examined for the presence of gas bubbles in the medium [17]. Production of gas indicated a positive reaction.

### *Lactobacillus *strains and culture conditions

27 *Lactobacillus *strains belonging to 8 different species were employed in this work and examined for their anti-microbial activity against coliforms isolated from colicky infants (Table 2). They were obtained from American Type Culture Collection, Manassas, VA, USA (referred to as ATCC strains), German Collection of Microorganisms and Cell Cultures, Braunschweig, Germany (referred to as DSM strains), National Collection of Dairy Organisms, Reading, England (referred to as NCDO strains) and from our collection (Department of Pharmaceutical Sciences, University of Bologna, Italy referred to as MB or S strains).

**Table 2:** *Lactobacillus *strains tested for their antagonist activity against coliforms isolated from colicky infants

Lactobacillus species	Strains
*L. acidophilus *	ATCC 11975; MB 252; MB 253; MB 358; MB 359; MB 422; MB 423; MB 424; MB 425; MB 442; MB443
*L. curvatus *	MB 67; MB 68
*L. casei *	ATCC 393; MB 50; MB 441
*L. delbrueckii *subsp.*delbrueckii *	DSM 20074
*L. delbrueckii *subsp.*lactis *	DSM 20076
*L. delbrueckii *subsp.*bulgaricus *	MB 453
*L. salivarius *subsp.*salicinius*	ATCC 11742
*L. salivarius *subsp.*salivarius *	ATCC 11741
*L. gasseri *	MB 335
*L. helveticus*	S 36.2; S40.8
*L. plantarum *	ATCC 8014; NCDO 1193; MB 456

### Assessment of the antagonistic activity

The antagonistic activity of the selected *Lactobacillus *strains against the isolated coliforms was assayed by using both agar plates and liquid co-cultures of both strains.

#### - Antimicrobial activity on agar plates

In this assay both *Lactobacillus *spp. cells and *Lactobacillus *neutralized cell-free supernatants (NCS) were employed. Each *Lactobacillus *strain was grown in MRS broth for 48 h at 37°C in 5% CO2 atmosphere and then centrifuged at 15000 *g *at 4°C for 15 minutes. pH of the cultures was neutralized to pH 7 with 1N NaOH and cells were separated through filtration (via a 0.2 μm pore size filter). *Lactobacillus *cells were washed twice with saline and suspended in saline at concentrations ranging from 10^4 ^to 10^6 ^CFU/ml. *Lactobacillus *cells were washed twice with saline and suspended in saline at concentrations of 10^4 ^, 10^5 ^and 10^6 ^CFU/ml. All the cell suspensions were assayed to optimize the most suitable cell concentration; the cell concentration of 10^6 ^CFU/ml was then used to perform the comparative assay of the inhibitory activity of the two *Lactobacillus *strains against coliforms. The paper-disk assay of Kirby-Bauer [23] was used with some modifications as follows. 50 μl of coliform liquid culture in LB broth containing from 10^3 ^to 10^6 ^CFU/ml, in the majority of cases between 10^5 ^and 10^6^, was streaked on a Mac Conkey and LB agar plate; subsequently two sterile paper blank disks (diameter 6 mm) were placed on the agar plate and imbibed one with 50 μl of washed *Lactobacillus *cells and the other with 50 μl of the corresponding NCS. After incubation for 18 h at 37°C, the diameters of the inhibition zones were evaluated. The experiments were made in triplicate.

#### - Antimicrobial activity in liquid co-cultures

The capability of *Lactobacillus *DSM 20074 of interfering with the growth of coliforms was evaluated by co-incubating both strains. The *Lactobacillus *strains and the coliform strains were grown on MRS broth and LB broth, respectively. The co-culture experiments was performed in a modified LB medium (i.e. LB additioned with 3% w/v yeast extract) capable of sustaining the growth of both microorganisms. The medium was inoculated with 10^5 ^CFU/ml of both the *Lactobacillus *and the coliform strains and incubated at 37°C in microaerophylic conditions. Controls were prepared by inoculating the same medium either with the *Lactobacillus *strain or with the coliform one; in addition coliforms were co-cultured with a *Lactobacillus *strain with no inhibition activity (*L. casei *MB50, Table 2). At 8-10 h intervals, cultures were centrifuged for 15 min at 5000 *g *and pellets were resuspended in fresh modified LB medium to limit changes in growth due to pH variation or nutrient limitation. 24 and 48 h after inoculation, bacterial cells were collected and thoroughly resuspended by vortexing in phosphate-buffered saline (PBS). Thereafter, *Lactobacillus *and coliform concentrations in the co-cultures and in the controls was determined on MRS agar plates additioned with vancomycin (0.2% w/v) and MacConkey agar plates, which are selective for *Lactobacillus *spp. and coliforms, respectively. Antimicrobial activity was calculated by comparing the coliform growth in the co-culture and control [8]. Results were expressed as log_10 _CFU/ml. The experiment was performed in triplicate.

### Statistical Analyses

Sample size was calculated based on a difference between groups of 1.5 log10 CFU/g faeces. Using α = 0.05, β = 0.20 and an estimated standard deviation within groups of 2 log10 CFU/g faeces, 30 patients were needed in each group. Counts (log10 CFU/g) of the total amount of coliform bacteria were calculated for each stool sample. Data are summarized by counts and median and range for categorical and continuous variables respectively. Differences between groups were evaluated with Mann-Whitney's U-test for continuous variables, whereas associations between categorical variables were evaluated with Fisher's exact test. Differences between colicky infants and controls in total amount of each species detected were evaluated with Mann-Whitney's test with Bonferroni correction. Statistical significance was set at a p-value < 0.05. All statistical calculations were performed with commercially available software (SPSS for Windows release 15Æ0 SPSS Inc., Chicago, IL, USA).

## Results

### Isolation and identification of coliforms from colicky infants

Coliform colonies were obtained on MacConkey agar plates from faeces of all the 45 colicky infants and 42 controls. The average count of total coliforms in the 45 faecal samples of colicky infants was 5.98 (2.00-8.76) log_10 _CFU/g of faeces, whereas total coliforms in the control group were 3.90 (2.50-7.10) log_10 _CFU/g of faeces. The difference between the two groups was statistically significant (p = 0.015). A total of 145 colonies was randomly picked up from the higher dilutions agar plates (10^-6^-10^-8^) and, only from colicky infants after sub-culturing in LB agar, each purified strain was examined for gas production and characterized at species level by DNA sequencing and carbohydrate fermentation profiling. All isolated strains were found to produce gas from lactose according to the method described above and the BBL™ Enterotube™ II system. They were ascribed to six different species (*Escherichia coli*, *Klebsiella oxytoca, Klebsiella pneumoniae*, *Enterococcus faecalis, Enterobacter aerogenes*, and *Enterobacter cloacae*), as described in Table 3. The percentage of detection of each species in the faecal samples examined was reported in descending order (Table 3). The same taxonomic identification was obtained with the two methods employed.

**Table 3:** Identification of the strains isolated from faeces of colicky infants at the species level and % of each species of the total colonies isolated from the faeces examined

Coliform identification	Quantitative detection (%)
*Escherichia coli *	55.45
*Klebsiella oxytoca*	22.15
*Klebsiella pneumoniae*	12.34
*Enterococcus faecalis *	6.20
*Enterobacter aerogenes*	2.70
*Enterobacter cloacae*	2.50

### Antimicrobial activity of lactic acid bacteria against coliforms

**Figure 1:**
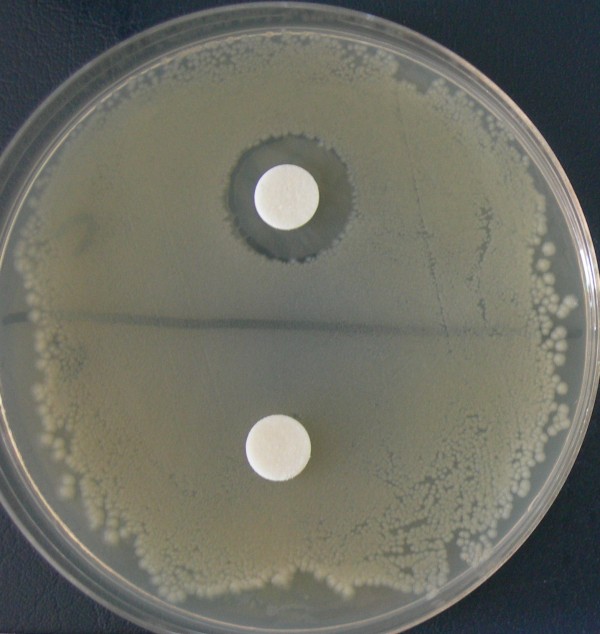
**Inhibitory activity of *L. delbrueckii *DSM 20074 against *E. coli *CG 15b**. Upper paper disk was imbibed with 50 μl of *L. delbrueckii *washed cells, whereas bottom paper disk was imbibed with 50 μl of neutralized supernatant of the same strain

One strain belonging to each species of isolated coliforms was selected in order to assess the antimicrobial activity of the 27 *Lactobacillus *strains described in Table 2. The coliform strains were referred to as *E. coli *CG 15b, *K. pneumoniae *CG 23a, *K. oxytoca *CG Z, *E. aerogenes *CG W,*E. cloacae *CG 6a and *E. faecalis *CG J. The antagonistic activity was initially examined by using the agar plates method employing both the NCS and washed cells. None of the NCS from all the *Lactobacillus *strains was found to inhibit the growth of the coliform strains, whereas the washed cells of two strains, i.e. *L. delbrueckii *subsp.*delbrueckii *DSM 20074 and *L. plantarum *MB 456, were found to possess strong inhibitory activity against all 6 coliforms as evidenced by the size of the inhibition halo determined on the coliform plates (Table 4). *L. delbrueckii *DSM 20074 exhibited a higher anti-bacterial activity against all the coliforms than the MB 456 strain. An example of the halo evidenced on the coliform plates is presented for *L. delbrueckii *DSM 20074 (Figure 1).

**Table 4:** Antagonistic activity of *L. delbrueckii *DSM 20074 and *L. plantarum *MB 456 cell suspensions (10^6 ^CFU/ml) against coliforms isolated from colicky infants

Coliform strains	Average diameter of the inhibition halo in mm (average ± SD)	
	*L. delbrueckii *DSM 20074	*L. plantarum *MB 456
*E. coli *CG 15b	10.23 ± 1.29	8.33 ± 0.89
*K. oxytoca *GC Y	9.75 ± 1.06	7.75 ± 0.76
*K. pneumoniae *CG 23a	9.83 ± 1.04	9.83 ± 0.64
*E. faecalis *GC W	10.16 ± 0.76	8.16 ± 0.56
*E. aerogenes *GC K	10.25 ± 0.65	7.25 ± 0.25
*E. cloacae *CG 6a	10.25 ± 0.35	7.05 ± 0.35

It has been expressed as average diameter of inhibition halos obtained on LB agar plates inoculated with each of the selected coliform strains

The anti-microbial activity evaluation in liquid co-cultures was performed with the *Lactobacillus *strain showing the highest anti-microbial activity with the previous method, i.e. *L. delbrueckii *subsp.*delbrueckii *DSM 20074, and each of the strains referred to the six species of coliform found. Inhibitory activity was evidenced against all the six coliform strains, being higher with the *E. coli *CG 15b strain. Referring to the experiment with DSM 20074 and *E. coli *CG 15b strains, the co-culture at the beginning of the incubation time contained 5.43 ± 0.54 log_10 _CFU/ml of *L. delbrueckii *DSM 20074 and 5.70 ± 0.35 log_10 _CFU/ml of *E. coli *CG 15b. After 24 h of incubation, the DSM 20074 concentration was increased to 9.84 ± 0.94 log_10 _CFU/ml, whereas no variations were observed in the *E. coli *count. In the parallel control experiment, in which *E. coli *was cultivated with no other strain, the *E. coli *concentration was 5.65 ± 0.34 and 9.00 ± 1.00 log_10 _CFU/ml at the beginning of the incubation and after 24 hours, respectively. When *E. coli *was co-cultured with *L. casei *MB50, no inhibition of *E. coli *growth was observed. In the co-culture experiments performed with *L. delbrueckii *DSM20074 and the other coliform strains listed in Table 3, an inhibition of the coliform growth of 3-4 log_10 _CFU/ml was observed (data not shown). On the other hand, the growth of the *Lactobacillus *strain was never influenced by co-cultivation with the coliform strains.

## Discussion

Different studies suggested that colonic gas production favours infantile colic, however the speculation is not supported by well-built scientific researches. Recently, it has been evidenced that gas forming coliform concentration is higher in colicky infants than in healthy controls [16]. Various medical interventions have already been applied to improve symptoms related to infantile colic. Simethicone, a defoaming agent, has been promoted as an effective treatment reducing the formation of intraluminal gas, even though existing data do not demonstrate conclusive benefit of such therapy [24,25]. Alternative solutions to the problem are therefore looked forward.

Recently the benefit of supplementation with *Lactobacillus reuteri *(American Type Culture Collection Strain 55730 and DSM 17 938) has been reported opening a new therapeutic approach [14,15], even though clinical trials are needed to promote new treatments to reduce abdominal pain related to infantile colic [16].

Coliform growth and carbohydrate fermentation affect ammonia absorption and urea nitrogen recycling and excretion. We observed reduction in fecal ammonia concentrations in breastfed infants given *L. reuteri *and this could be related to modification of bacterial enzyme activity depending on gut microbiota and suggested that gas forming coliforms may be involved in determining colonic fermentation and consequently excessive intraintestinal air load, aerophagia and pain, characteristic symptoms of colic crying, but many aspects of these relationships are still unclear [15]. In the present study we confirmed the higher count of coliforms in colicky infants with respect to non colicky newborns, as already observed in a previous work [17].

Previous studies had shown that some *Lactobacillus *spp. strains possessed inhibitory activity against *E. coli*, preventing the binding of enteropathogenic *E. coli *and other pathogens to intestinal cells [26]. More recently it has been shown that a synbiotic diet containing both prebiotics and probiotics reduces population of intestinal *E. coli *and the pathogen population in rats [27].

Given these findings, in this work new *Lactobacillus *strains possessing anti-microbial activity against gas-producing coliforms were searched and the interaction between selected lactobacilli and coliforms was studied.

Coliforms were isolated from stools of colicky infants and characterized taxonomically and for gas production. They were all gas-producing strains and were attributed to 6 different species. The taxonomic identification of the isolated strains and their relative percentage within the coliform group confirmed the results obtained in a previous study, being *E. coli *the most represented species [17]. Two of the 27 lactic acid bacteria assayed in this study, *L. delbrueckii *subsp.*delbrueckii *DSM 20074 and *L. plantarum *MB 456, were able to inhibit the growth of gas-forming coliforms belonging to the different species isolated from colicky infants. The extent of the inhibitory activity was similar for all the coliforms assayed (Table 4), although it was higher for the DSM 20074 strain with respect to the other one. Moreover, the capability of the DSM 20074 strain of hindering the growth of coliforms was also observed in a liquid co-culturing assay. Therefore, this strain appears to be a good candidate to relieve symptoms caused by gas-producing coliforms in colicky infants.

The antagonistic activity of the two *Lactobacillus *strains was only evidenced when harvested cells were applied, whereas the neutralized culture supernatants did not exert any activity on the same coliforms (Figure 1). The inhibitory activity of lactic acid bacteria has generally been ascribed to two mechanisms, which can often coexist: i) the production of bacteriocins or bacteriocin-like molecules, which are very often secreted outside the cell [28,29] and ii) the production of inhibitory non proteinaceous metabolites such as organic acids, carbon dioxide, ethanol, hydrogen peroxide and diacetyl, whose anti-microbial action is well known [30]. In addition, Alakomi et al. reported that lactic acid can permeabilize the membrane of Gram negative bacteria by a mechanism of outer membrane disruption [31]. In the case of the two lactic acid bacteria showing inhibitory activity against coliforms in this work, this activity is linked to the presence of the whole cells, although it is not possible to exclude that putative inhibitory molecules are present in the supernatants at such a low concentration that their activity cannot be detected by the assay employed. Therefore, it is not possible to clearly ascribe the inhibitory activity to a defined group of molecules and further studies are necessary to characterize the exact mechanism of inhibition.

## Conclusions

In conclusion, this study confirmed the presence of a greater amount of coliforms in colicky infants with respect to the controls, mainly belonging to the *E. coli *species.

*L. delbrueckii *subsp.*delbrueckii *DSM 20074 strain expressed potential for the inhibition of gas-producing coliforms, and thus can be considered a promising candidate for the treatment of colicky symptoms in infants. At present, few data are available on the role of probiotics in colic and the mechanisms by which probiotic bacterial strains antagonise pathogenic gastrointestinal microorganisms or exert other beneficial effects *in vivo *have not yet been fully defined. Even so, there is a growing interest within clinical medicine in the understanding of the mechanisms through which lactic acid bacteria exert their antagonistic activity against pathogens in the gut. Finally, clinical investigations about *in-vivo *efficacy are necessary to confirm the role of *Lactobacillus *strains as efficacious probiotic treatment to modulate the colonic microbiota in newborns and improve abdominal discomfort due to infantile colic.

## Competing interests

The authors declare that they have no competing interests.

## Authors' contributions

FS had primary responsibility for the paper and drafted the manuscript. LC performed the molecular analyses. VT and EL were responsible for the screening of patients, enrolment and outcome assessment. DDG performed the microbiological analyses. RO had primary responsibility for patients enrolled. DM conceived all the study, participated in its design and coordination and helped to draft the manuscript. All authors read and approved the final manuscript.

## References

[B1] Guarner F Enteric flora in health and disease Digestion 2006 73 5 12 10.1159/000089775 16498248

[B2] Sjögren YM Tomicic S Lundberg A Böttcher MF Björkstén B Sverremark-Ekström E Jenmalm MC Influence of early gut microbiota on the maturation of childhood mucosal and systemic immune responses Clin Exp Allergy 2009 39 1842 51 10.1111/j.1365-2222.2009.03326.x 19735274

[B3] Penders J Thijs C Vink C Stelma FF Snijders B Kummeling I van den Brandt PA Stobberingh EE Factors influencing the competition of intestinal microbiota in early infancy Pediatrics 2006 118 511 21 10.1542/peds.2005-2824 16882802

[B4] Adlerberth I Wold AE Establishment of the gut microbiota in Western infants Acta Paediatr 2009 98 229 38 10.1111/j.1651-2227.2008.01060.x 19143664

[B5] Miller JJ Mc Veagh P Fleet GH Petocs P Brand JC Breath hydrogen excretion in infants with colic Arch Dis Child 1989 64 725 9 10.1136/adc.64.5.725 2730128 PMC1792036

[B6] Lehtonen L Korvenranta H Eerola E Intestinal microflora in colicky and non-colicky infants: bacterial cultures and gas-liquid chromatography J Pediatr Gastroenterol Nutr 1994 19 310 4 10.1097/00005176-199410000-00009 7815263

[B7] Treem WR Infant colic: a pediatric gastroenterologist's perspective Pediatr Clin North Am 1994 41 1121 38 7936776 10.1016/s0031-3955(16)38848-4

[B8] Belson A Shetty AK Yorgin PD Bujanover Y Peled Y Dar MH Reif S Colonic Hydrogen elimination and methane production in infants with and without colic syndrome Dig Dis Sci 2003 48 1762 76 10.1023/A:1025595029795 14560998

[B9] Moore DJ Robb TA Davidson GP Breath hydrogen response to milk containing lactose in colicky and non-colicky infants J Pediatr 1988 113 979 84 10.1016/S0022-3476(88)80567-5 3193321

[B10] Miller JJ Brand JC McVeagh P Breath hydrogen excretion in infants with colic Arch Dis Child 1990 65 248 2317079 10.1136/adc.65.2.248-a PMC1792219

[B11] Kanabar D Randhawa M Clayton P Improvement of symptoms in infant colic following reduction of lactose load with lactase J Hum Nutr Diet 2001 14 359 63 10.1046/j.1365-277X.2001.00304.x 11906576

[B12] Savino F Cresi F Pautasso S Palumeri E Tullio V Roana J Silvestro L Oggero R Intestinal microflora in breastfed colicky and non-colicky infants Acta Paediatr 2004 93 825 9 10.1111/j.1651-2227.2004.tb03025.x 15244234

[B13] Savino F Bailo E Oggero R Tullio V Roana J Carlone N Cuffini AM Silvestro L Bacterial counts of intestinal *Lactobacillus *species in infants with colic Pediatr Allergy Immunol 2005 16 72 5 10.1111/j.1399-3038.2005.00207.x 15693915

[B14] Savino F Pelle E Palumeri E Oggero R Miniero R *Lactobacillus reuteri *(American Type Culture Collection Strain 55730) versus simethicone in the treatment of infantile colic: a prospective randomized study Pediatrics 2007 119 e124 30 10.1542/peds.2006-1222 17200238

[B15] Savino F Cordisco L Tarasco V Palumeri E Calabrese R Oggero R Roos S Matteuzzi D *Lactobacillus reuteri *DSM 17938 in infantile colic: a randomized, double-blind, placebo-controlled trial Pediatrics 2010 126 e526 33 10.1542/peds.2010-0433 20713478

[B16] Savino F Tarasco V New treatments for infantile colic Curr Opin Pediatr 2010 22 791 797 10.1097/MOP.0b013e32833fac24 20859207

[B17] Savino F Cordisco L Tarasco V Calabrese R Palumeri E Matteuzzi D Molecular identification of coliform bacteria from colicky breastfed infants Acta Paediatr 2009 98 1582 8 10.1111/j.1651-2227.2009.01419.x 19604166

[B18] Jiang T Suarez FL Levitt MD Nelson SE Ziegler EE Gas production by feces of infants J Pediatr Gastroenterol Nutr 2001 32 534 41 10.1097/00005176-200105000-00009 11429513

[B19] Penders J Vink C Driessen C London N Thijs C Stobberingh EE Quantification of *Bifidobacterium *spp., *Escherichia coli *and *Clostridium difficile *in faecal samples of breast-fed and formula-fed infants by real-time PCR FEMS Microbiol Lett 2005 243 141 7 10.1016/j.femsle.2004.11.052 15668012

[B20] Wessel MA Cobb JC Jackson EB Harris GS Detwiler AC Paroxismal fussing in infancy, sometimes called "colic" Pediatrics 1954 14 421 35 13214956

[B21] Nakamura N Gaskins HR Collier CT Nava GM Rai D Petschow B Molecular ecological analysis of fecal bacterial populations from term infants fed formula supplemented with selected blends of probiotics Appl Environ Microbiol 2009 75 1121 8 10.1128/AEM.02359-07 19088307 PMC2643592

[B22] Sambrook J Fritsch EF Maniatis T Molecular Cloning: A Laboratory Manual 1989 2 New York: Cold Spring Harbor Laboratory Press

[B23] Bauer AW Kirby WMM Sherris JC Turck M Antibiotic susceptibility testing by a standardized single disk method Am J Clin Pathol 1966 45 493 6 5325707

[B24] Garrison MM Christakis DA A systematic review of treatments for infant colic Pediatrics 2000 106 184 90 10888690

[B25] Lucassen PL Assendelft WJ Gubbels JW van Eijk JT van Geldrop WJ Neven AK Effectiveness of treatments for infantile colic: systematic review BMJ 1998 316 1563 9 9596593 10.1136/bmj.316.7144.1563 PMC28556

[B26] Servin AL Antagonistic activities of lactobacilli and bifidobacteria against microbial pathogens FEMS Microbiol Rev 2004 28 405 40 10.1016/j.femsre.2004.01.003 15374659

[B27] Liong MT Shah NP Effects of a *Lactobacillus casei *synbiotic on serum lipoprotein, intestinal microflora, and organic acids in rats J Dairy Sci 2006 89 1390 9 10.3168/jds.S0022-0302(06)72207-X 16606710

[B28] Vandenbergh PA Lactic acid bacteria, their metabolic products and interference with microbial growth FEMS Microbiol Rev 1993 12 22 38

[B29] Santini C Baffoni L Gaggìa F Granata M Gasbarri R Di Gioia D Biavati B Characterization of probiotic strains: an application as feed additives in poultry against *Campylobacter jejuni* Int J Food Microbiol 2010 141 S98 S108 20452074 10.1016/j.ijfoodmicro.2010.03.039

[B30] Galvez A Abriouel H Benomar N Lucas R Microbial antagonists to food-borne pathogens and biocontrol Curr Op Biotechnol 2010 21 142 8 10.1016/j.copbio.2010.01.005 20149633

[B31] Alakomi HL Skytta E Saarela M Mattila-Sandholm T Latva-Kala K Helamder IM Lactic acid permeabilizes Gram-negative bacteria by disrupting the outer membrane Appl Environ Microbiol 2000 66 2001 5 10.1128/AEM.66.5.2001-2005.2000 10788373 PMC101446

